# Genotype-specific Distribution and Change of High-risk Human Papillomavirus Infection and the Association with Cervical Progression Risk in Women with Normal Pathology and Abnormal Cytology in a Population-based Cohort Study in China

**DOI:** 10.7150/jca.57993

**Published:** 2021-05-19

**Authors:** Haixia Jia, Ling Ding, Yang Han, Yuanjing Lyu, Min Hao, Zhiqiang Tian, Jintao Wang

**Affiliations:** 1 Department of Epidemiology, School of Public Health, Shanxi Medical University, Taiyuan, China.; 2 Department of Obstetrics and Gynecology, The Second Hospital of Shanxi Medical University, Taiyuan, China.

**Keywords:** high-risk human papillomavirus, genotype, normal cervix, progression, cohort

## Abstract

**Objectives:** Women with normal pathology screened from abnormal cervical cytology are a special population with higher progression risk than women with normal cytology. However, the associations between genotype distribution and changes of high-risk human papillomavirus (HR-HPV) infection and cervical progression risk in this special population remain unclear.

**Methods:** A total of 1232 women with normal pathology screened from abnormal cervical cytology were enrolled into this cohort with 2-year follow-up. HPV genotyping detection was performed through flow-through hybridization. Hazard ratios (HRs) and Odds ratios (ORs) were calculated using Cox proportional hazard regression and logistic regression models, respectively.

**Results:** Overall HR-HPV prevalence at baseline was 29.0%, with HPV16, 52, 58, 53 and 51 the top five genotypes. The 2-year persistence rate of HR-HPV infection was 31.9%. Compared with HR-HPV negative, the adjusted HRs of overall HR-HPV, HPV16, 31/33, 58, 51, and 53 infections for the progression risk of normal cervix were 5.31, 7.10, 6.95, 5.74, 5.04, and 4.88, respectively. Multiple HR-HPV infection cannot lead to an additional risk of progression relative to single HR-HPV infection. In comparison with HR-HPV persistently negative, same-type HR-HPV persistence was positively associated with progression risk of normal cervix (adjusted OR: 22.26), but different-type HR-HPV persistence was not linked to cervical progression.

**Conclusion:** Genotypes and persistence of HR-HPV infection could stratify the cervical progression risk in women with normal cervical pathology and abnormal cytology and provide evidence for development of next generation of vaccines. HPV51 and 53 deserved attention apart from HPV16, 31, 33, and 58.

## Introduction

Cancers caused by Human papillomavirus (HPV) infection, including cancers of the cervix, anus, vulva, vagina, penis and oropharynx and so on, account for approximately 4.5% of all cancers globally 1. Cervical cancer accounts for 83% of HPV-attributable cancers, with about 570,000 women newly diagnosed with cervical cancer and 311,000 cases dying from cervical cancer each year worldwide 1,2. China has approximately 102,000 new cases and 31,000 deaths of cervical cancer annually 3. Thus, the burden of cervical cancer remains an important public health problem threatening women's physical health.

The causal role of HPV infection, especially high-risk HPV (HR-HPV) infection has been established in cervical carcinogenesis by previous studies 4,5. More than 100 of HPV genotypes have been identified from humans to date 6. They are categorized as HR-HPV genotypes and low-risk HPV (LR-HPV) genotypes according to their carcinogenesis. However, the oncogenic potential of HR-HPV infection in cervical carcinogenesis varies with genotypes. HPV16, the most carcinogenic genotype globally, has been used to predict the risk of cervical cancer and its precursors in the general population 7-10. HPV18 has attracted special attention in western countries due to its high prevalence and high carcinogenicity 7,8. HPV31/33 infections have also been reported to promote cervical carcinogenesis worldwide 7-10. HPV52 and 58 are the genotypes with oncogenic potential and high prevalence in Asia regions 10,11. The 5-year risk for cervical intraepithelial neoplasia grade 2 and above (CIN2+) was much higher (45.3%) in women with mild abnormal cervical cytology than that (12.9%) in women with normal cervical cytology 12, suggesting that women with normal pathology screened from abnormal cervical cytology were a special population with higher progression risk than women with normal cytology. However, the effect of HR-HPV genotyping in cervical progression and the associations between changes of HR-HPV infection and cervical progression remain unclear in this special population.

In this CIN screening cohort study, we aimed to describe the genotype distribution and changes of HR-HPV infection and evaluate the relationship between them and the progression risk of pathologically normal cervix screened from abnormal cytology, in order to provide evidence for risk stratification of women with normal pathology and abnormal cytology in Chinese and for the development of next generation of vaccines with broader coverage of significant genotypes.

## Materials and methods

### Study population and design

**Figure 1:**
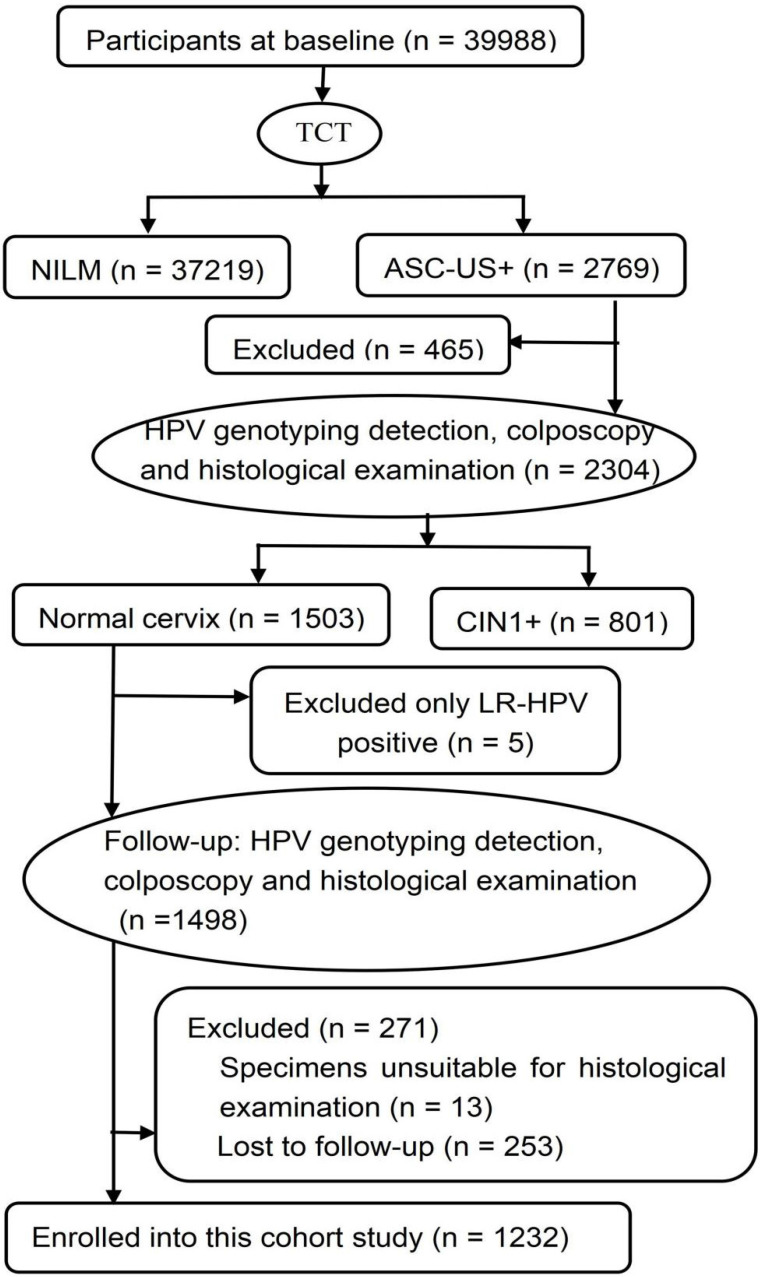
Flow chart of participants in this cohort study. TCT: thinprep cytologic test; NILM: negative for intraepithelial lesion or malignancy; ASC-US+: atypical squamous cells of undetermined significance and above; HPV: human papillomavirus; CIN1+: low-grade cervical intraepithelial neoplasia and above; LR-HPV: low-risk HPV.

A CIN community cohort study including 39,988 women aged 18-65 years was established in two counties (Jiexiu and Yangqu) in Central Shanxi Province between June 2014 and December 2014. Baseline screening procedures have been described in detail previously 13. All 39,988 women completed a uniform socio-demographic and characteristic-related questionnaire and thinprep cytologic test (TCT). A total of 2769 women were diagnosed with atypical squamous cells of undetermined significance and above (ASC-US+) by TCT. Of the 2769 women with ASC-US+, 2304 women completed HPV typing detection, colposcopy-directed punch biopsy and histological examination. Of the 2304 women who underwent pathological examination, 1503 women were diagnosed with normal cervix. Of the 1503 women with normal pathology, 5 women with only LR-HPV positive were excluded, 271 women were excluded at follow-up, including 13 whose specimens were unsuitable for histological examination, 253 women who were lost to follow-up. The remaining 1232 women, in the same turn in which they were originally enrolled, completed in-person interviews, HPV typing detection, colposcopy and histological examination again 2 years after enrollment (from June 2016 and December 2016). A total of 1232 eligible women were finally enrolled in this study (**Figure 1**). All of the enrolled participants were married, Han ethnicity, Shanxi native, non-pregnant, and had no history of HPV vaccination, cervical and vaginal lesion treatments, hysterectomy, and other malignant tumors. Written informed consents were signed by all women and the study was approved by Shanxi Medical University Science Research Ethics Committee. (No: 2013-003).

### Baseline data collection

Information of age, smoking, drinking, education level, yearly income, medical history, age at first intercourse, contraception history, parity, menopause history, hygiene habits, and family history of cancer was collected using a uniform structured questionnaire from an in-person interview. Information of HPV typing and pathological diagnoses was collected from laboratory tests.

### Specimen collection

After removing the cervical secretions with a cotton swab, exfoliated cervical cells were collected using a HPV special cervical brush. Cervical biopsy tissues were collected under colposcopy (Shenzhen Goldway Industrial Co. Ltd., China) according to the Preventive Oncology International microbiopsy protocol.

### Laboratory tests

#### Pathological examination

Pathological diagnoses were completed together by two gynecologic pathologists using cervical biopsy tissues. If the two pathologists reached different diagnoses, a third senior pathologist was invited to discuss the difficult or equivocal cases to arrive at a consensus diagnosis. In addition, immunohistochemical detection of p16 and Ki-67 was performed to assist the pathological diagnosis. Pathological results were classified as normal cervix, low-grade CIN (CIN1), high-grade CIN (CIN2/3), and cervical squamous cell carcinoma.

### HPV genotyping detection

HPV genotyping testing was performed using the 21 HPV GenoArray Diagnostic Kit (HybriBio Co. Ltd., Chaozhou, China) using exfoliated cervical cells. Compared with standard Roche Linear Array and hybrid capture II, most HPV genotypes detected with this kit shows good interassay agreement 14,15. Specific experimental procedures have been described in detail in our previous study 16. Exfoliated cervical cells were centrifuged at 4°C, 13000 rpm for 15min, to obtain the precipitate. Then, HPV-DNA was extracted from the precipitate using a DNA extraction kit. A PCR-based flow-through hybridization and gene chip system were used for HPV hybridization and color rendering. Purple and blue dots were regarded as HPV positive by the HPV genotypes schematic diagram. This assay can identify 15 HR-HPV genotypes (HPV16, 18, 31, 33, 35, 39, 45, 51, 52, 53, 56, 58, 59, 66, and 68) and 6 LR-HPV genotypes (HPV6, 11, 42, 43, 44, and CP8304). HR-HPV infection meant that at least one HR-HPV genotype was positive. Infection with only one HR-HPV type was referred to as single HR-HPV infection. Infection with two or more HR-HPV types was regarded as multiple HR-HPV infection. Single or mixed infection of a certain HR-HPV genotype was considered as this subtype infection. In cases of coinfection with different HR-HPV genotypes, the same patient can be included in the statistical analysis of multiple HR-HPV categories.

### Follow-up

From June 2016 and December 2016, 2 years after enrollment, all women with normal pathology screened from abnormal cervical cytology were followed up except for only LR-HPV positive women. The follow-up contents included an in-person interview, HPV typing detection, colposcopy and histological examination. The outcomes of follow-up included the prognosis of normal cervix and the changes of HR-HPV infection. Persistent HR-HPV infection (persistence), including persistent same-type HR-HPV infection and persistent different-type HR-HPV infection, referred to HR-HPV positive at baseline and follow-up. Persistent same-type HR-HPV infection meant that at least one HR-HPV genotype was identical at baseline and follow-up; persistent different-type HR-HPV infection meant that none of the HR-HPV genotypes detected at follow-up was the same as those detected at baseline. The change from HR-HPV positive to negative was considered as the clearance of HR-HPV. Conversely, the change from HR-HPV negative to positive was regarded as the acquisition of HR-HPV. The prognosis of normal cervix included progression (transiting to CIN1 and above (CIN1+)) and persistence (persisting as normal cervix).

### Statistical analyses

The SPSS 22.0 software was used for data statistical analysis. Categorical variables were presented as frequency or proportion and continuous variables as mean and standard deviation. We used chi-square test to examine the differences among different groups. Hazard ratios (HRs) and 95% confidence intervals (CIs) were calculated using Cox proportional hazard regression models to present the effect of baseline HR-HPV genotype-specific infection in progression of normal cervix. Odds ratios (ORs) and 95% CIs were estimated using binary unconditional logistic regression models to present the associations between changes of HR-HPV infection and progression of normal cervix. Statistical significance was set at α = 0.05 on the basis of 2-sided tests.

## Results

### Baseline characteristics

We examined the distribution of demographic characteristics between the 1232 participants and the 271 women who were excluded at follow-up and found no significant difference (**Table 1**). The mean ages of the 1232 women enrolled into this cohort were 48.73 ± 8.56 years (range 20-65 years). Of the 1232 participants, 1025 (83.2%) women had completed middle school or above, 20 (1.6%) women smoked, 52 (4.2%) women drank alcohol, 882 (71.6%) women had an income of more than 10,000 ¥RMB annually, 409 (33.2%) women had a history of female sterilization, 112 (9.1%) women had a family history of cancer, 463 (37.6%) women's age at first intercourse was under 23 years old, 82 (6.7%) women had a history of oral contraceptive use, 303 (24.6%) women's parity was 3 or more and 608 (49.4%) women had a history of menopause.

### Genotype distribution and changes of HR-HPV infection

**Figure 2:**
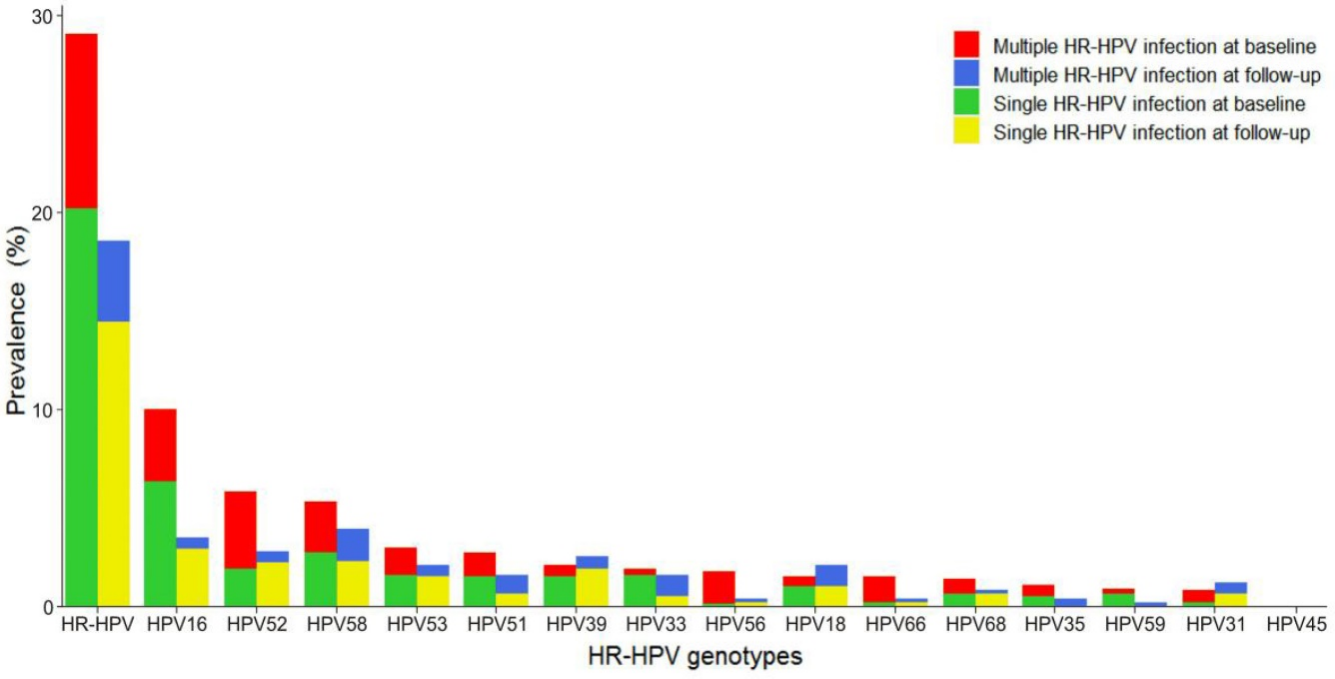
Distribution of genotype-specific HR-HPV infection. HR-HPV: high-risk human papillomavirus.

The distribution of various HR-HPV genotypes differed, as shown in **Figure 2**. Overall HR-HPV prevalence was much lower at follow-up (18.4%) than at baseline (29.0%). Single HR-HPV prevalence, either at baseline or follow-up, was much higher than that of multiple HR-HPV. The most common HR-HPV genotype was HPV16 at baseline and HPV58 at follow-up. However, HPV16, 52, 58 were the three most frequently observed genotypes in this screening sample at baseline and follow-up. HPV16 and 58 occurred mainly as single infection. HPV52 occurred mainly as multiple infection at baseline while single infection at follow-up. HPV53, 51, 39, and 33 were also common at baseline and follow-up, with HPV39 and 53 mainly in the form of single infection and HPV33 and 51 mainly in the form of single infection at baseline and multiple infection at follow-up.

Compared with baseline, overall clearance and persistence rates of HR-HPV infection at 2-year follow-up were 68.1% (243/357) and 31.9% (114/357), respectively. Of the 875 women with HR-HPV negative at baseline, 113 (12.9%) turned positive for HR-HPV and 762 (87.1%) remained negative for HR-HPV at follow-up. Of the 114 women with HR-HPV persistence, 67 (58.8%) were persistent same-type infection and 47 (41.2%) were persistent different-type infection.

### Prognosis of normal cervix and related baseline factors affecting progression of normal cervix

Of the 1232 participants, 22 (1.8%) progressed to CIN1, 10 (0.8%) progressed to CIN2/3, and 1200 (97.4%) persisted still as normal cervix at follow-up. Therefore, a total of 32 (2.6%) women progressed. Between the progression and persistence groups of normal cervix, there were statistically significant differences in oral contraceptive use (χ^2^ = 5.87, *p* = 0.015) and age (χ^2^ = 8.93, *p* = 0.010), while there was no significant difference in the distribution of yearly income, education level, smoking, drinking, menopause history, parity, age at first intercourse, frequency of vulva cleaning, frequency of underwear cleaning, history of female sterilization, and family history of cancer (*p* > 0.05). Women aged 20-35 years old and those who had used oral contraceptives for more than 1 year were more likely to progress.

### Effect of baseline HR-HPV genotype-specific infection in progression of normal cervix

**Table 2** shows the effect of baseline HR-HPV infection in the progression of normal cervix through the use of Cox proportional hazard analyses. After full adjustment including oral contraceptive use and age, we observed that HR-HPV infection (adjusted HR (aHR = 5.31; 95% CI: 2.51-11.21), either single HR-HPV infection (aHR = 4.67; 95% CI: 2.07-10.55) or multiple HR-HPV infection (aHR = 4.67; 95% CI: 1.74-12.61), significantly increased the risk for the progression of normal cervix in relative to HR-HPV negative group. Compared with women with HR-HPV negative, the aHRs for the progression risk of normal cervix were 7.10 (95% CI: 3.05-16.53) for HPV16 infection, 6.95 (95% CI: 2.01-26.26) for HPV31/33 infection, 5.74 (CI: 1.94-16.96) for HPV58 infection, 5.04 (95% CI: 1.06-23.94) for HPV51 infection, and 4.88 (95% CI: 1.06-22.43) for HPV53 infection. We did not found significant associations between HPV18, 35, 39, 52, 56, 59, 66, or 68 infections and the progression of normal cervix.

**Table 3** compares the role of single and multiple HR-HPV infections in the progression of normal cervix by using Cox proportional hazard analyses. Compared with single HR-HPV infection, multiple HR-HPV infection (aHR = 1.21; 95% CI: 0.48-3.04) did not lead to a significant increased progression risk for normal cervix. Meanwhile, no significant differences associated with the risk for the progression of normal cervix were observed between single infection and multiple infection of HPV16, 31/33, 51, 53, or 58.

### Association between changes of HR-HPV infection and progression of normal cervix

**Table 4** displays the associations between the changes of HR-HPV infection and the risk of cervical progression among the 1232 women with normal histology but abnormal cervical cytology through the use of binary unconditional logistic regression analyses. After fully adjusting for oral contraceptive use and age, we found that persistent HR-HPV infection (aOR = 13.96; 95% CI: 5.71-34.10), especially persistent same-type HR-HPV infection (aOR = 22.26; 95% CI: 8.98-55.17), was a risk predictor for the progression of normal cervix. No significant difference was observed in the cervical progression risk between the HR-HPV persistently negative group and the HR-HPV clearance group or the HR-HPV acquisition group. In addition, we also compared the cervical progression risk between the HR-HPV persistence group, especially the persistent same-type HR-HPV infection group, and other groups. Compared with the HR-HPV clearance group and the new acquisition group, the aORs for the progression risk of normal cervix in the HR-HPV persistence group were 6.64 (95% CI: 2.52-17.52) and 5.00 (95% CI: 1.46-17.19), respectively, and the aORs for the cervical progression risk in the persistent same-type HR-HPV infection group were 8.21 (95% CI: 3.06-23.01) and 7.19 (95% CI: 2.05-25.21), respectively. In relative to the persistent different-type HR-HPV infection group, the aOR for the progression risk of normal cervix in the persistent same-type HR-HPV infection group was 4.61 (95% CI: 1.10-20.31).

## Discussion

HPV prevalence is dramatically different by geographical region and increase notably with the severity of cervical lesions. In the present study, overall HR-HPV prevalence was 29.0% at baseline, higher than 18.2% HPV prevalence in Xinjiang, China 17, but far lower than 66.67% HPV prevalence in Tianjin, China 18. Although the subjects were all pathologically normal women screened from abnormal cervical cytology, the studies in XinJiang and Tianjin included much smaller objects than our study, only 40 and 33 women, respectively, which severely restricted their statistically performance. Additionally, 29.0% overall HR-HPV prevalence at baseline and 18.4% at follow-up in this cohort were higher than average HPV prevalence (10.4%) in women with normal cytology globally 19 and HPV prevalence (11.2%) of Chinese women with normal cervical cytology/histology as reported in the meta-analyses 20. The reasons for the differences in HPV prevalence may be as follows. First, the research objects were different. Our participants were women with pathologically normal cervix screened from women with abnormal cytology. Globally, the HR-HPV prevalence in women with cervical cytological abnormality (69.8%) was significantly higher than that (12.0%) in women with cervical cytological normality 4. Second, HPV prevalence varied among different populations. It has been reported that the HR-HPV prevalence in women with cervical abnormal cytology was 52.3%, 70.8, and 85.3% in Africa, Western/Central Asian, and Oceania, respectively 4.

In our cohort, HPV16 was the most prevalent HR-HPV genotype at baseline, followed by HPV52, 58, 53, 51, 39, 33, 56, 66, 18, 68, 35, 59, and 31. HPV16 was the most common HPV type, which was consistent with those reported in previous studies worldwide 19,21. The most prevalent HR-HPV genotype was HPV58 at follow-up, followed by HPV16, 52, 39, 18, 53, 33, 51, 31, 68, 35, 56, 66, and 59. These results demonstrated that HR-HPV genotype distribution varied at baseline and follow-up in this screening sample. However, HPV16, 52, and 58, either at baseline or follow-up, were the top three HPV genotypes, which was aligned with the results in several studies of Chinese women 10,22,23. HPV18, highly prevalent globally, accounted for a low proportion in our cohort. HPV45, commonly seen in South America, Southern Europe and Northern Africa 19, was not detected in our cohort. Similarly, the prevalence of HPV18 was not high and the prevalence of HPV45 was particularly low in a CIN cohort study in Xiangyuan, Shanxi Province 10. These deviations might be linked to the complex biological interplay between HPV subtypes and host immune response in various geographical regions. Our study found that the prevalence of single HR-HPV infection (69.7% at baseline and 77.8% at follow-up) was higher than that of multiple HR-HPV infection (30.3% at baseline and 22.2% at follow-up), which was similar with the results in several other studies in China 18,24,25. In those studies, the prevalence of single HPV infection ranged from 62.8% to 92.28%. HPV 16, 58, and 52, either in multiple HR-HPV infection or in single HR-HPV infection, were the top three most observed genotypes at baseline. Our results showed that the 2-year clearance and acquisition probabilities of HR-HPV were 68.1% and 12.9%, respectively. Our results were consistent with those in previous studies that the majority of HPV infection was cleared and about 10% of HR-HPV infection was newly acquired within 2 years 26-28. Although the 2-year clearance rate of HR-HPV was high, there were still a considerable number of women with persistent HR-HPV infection. Persistent HR-HPV infection has been regarded as the main cause for development of cervical cancer 4,5. The 2-year persistence rate of HR-HPV was 31.9% in our study, posing a high burden for progression of normal cervix. In a retrospective cohort study in Guangdong, China, the 2-year persistence rate of HR-HPV in outpatients was even as high as 42.77% 29. In our cohort, more than half (58.8%) of women with persistent HR-HPV infection were persistently infected with the same-type of HR-HPV genotypes. Previous studies rarely distinguish between persistent same-type and different-type HR-HPV infection.

The occurrence of cervical cancer has a “classic” process, from normal cervix, through CIN1, to CIN2/3, and finally to cervical cancer. In this study, the 2-year progression rate of normal cervix to CIN1+ was 2.6%, posing a risk for subsequent invasive cervical cancer. Our analysis results showed that women aged 20-35 and those who had used oral contraceptives for more than 1 year were more likely to develop CIN1+. Oral contraceptive use has been reported to increase the risk of developing cervical cancer in women with normal cytology in a retrospective population-based cohort study 30. Recently, a UK prospective cohort study found that the progression probability of normal cervical cytology to CIN2+ decreased with age, with the highest in the 25-34 years old population 31.

HR-HPV infection is widely known as the major cause of cervical lesion progression 32.We found that the progression rate in HR-HPV positive women was 5.39 times of that in women with HR-HPV negative. The cervical progression risk varied markedly with different HR-HPV subtypes 7-10. We fully adjusted for the effect of oral contraceptive use and age on progression of normal cervix in the current cohort. Our study results indicated that, compared with HR-HPV negative, HPV16, 31, 33, 51, 53, and 58 infections increased the progression risk of normal cervix, while HPV18, 35, 39, 45, 52, 56, 59, 66, and 68 infections were not found to be correlated with cervical progression. These results clearly suggested that we could stratify more precisely the progression risk in women with normal pathology and abnormal cervical cytology by genotype-specific HR-HPV testing than HPV testing that did not distinguish individual types. The increased risk for the progression of normal cervix from HPV16 infection was the greatest, followed by HPV31/33, 58, 51 and 53 infections. HPV16 and 31/33 were the top three genotypes with the highest risk of cervical progression, which was consistent with the results in a Sweden cohort study with 4-year follow-up and a CIN cohort study in Xiangyuan, Shanxi Province with 15-year follow-up 9,10. Two Denmark cohort studies with a maximum follow-up period of up to 11 years also found that the cervical progression risk in HPV16 positive women was the highest, followed by HPV18, 31, and 33 8,33. HPV58 infection was worthy of notice for an increased cervical progression risk, as well as for the high prevalence in Asian population, as revealed by our study and previous studies 10,34. HPV51 infection has been reported to increase the cervical progression risk 7,9, which was also found in our cohort. In addition, we found that HPV53 infection led to an increased cervical progression risk with rare report in previous studies. At least 26 genomic variants of HPV53 have been found, which may play different roles in cervical progression in different populations 35. HPV52 was the second most common genotype, while no association was observed between its infection and cervical progression in our cohort. HPV18, a genotype with the second highest cervical progression risk in some western countries 7,33, did not increase the cervical progression risk in our cohort. However, HPV18 positive women had much higher 10-year CIN2+ risk (12.0%) than HR-HPV negative women (2.7%) in the Xiangyuan CIN cohort study 10. This difference may be related to different population and follow-up time. HPV16, 31, 33, and 58, strongly associated with the progression of pathologically normal cervix screened from abnormal cytology, have been targeted by 9-valent HPV vaccines (HPV6, 11, 16, 18, 31, 33, 45, 52, and 58 genotypes) 36. We found that HPV51 and 53 infections were not only common, but also increased the progression risk of normal cervix. However, the current HPV vaccines did not covered HPV51 and 53 genotypes, suggesting that the next generation of vaccines might need to cover these genotypes for cervical lesion prevention. Women with HPV16, 31/33, 58, 51, and 53 positive were at high progression risk and needed close follow-up to detect early cervical lesions.

Compared with single HR-HPV infection, the role of multiple HR-HPV infection in cervical progression is controversial. Most cohort studies have shown that multiple HPV infection offers no additional risk than single HPV infection 10,34,37. However, some studies have found that multiple HPV infection lead to an increased risk of cytology abnormality compared to single HPV infection 38,39. We found that multiple HR-HPV infection, either overall HR-HPV, HPV16, 31, 33, 51, or 58, could not increase the cervical progression risk in comparison to single HR-HPV infection, which might be due to the competitive growth of HPV genotypes. When one major HPV genotype proliferates, other types of HPV are in infection dormant state 5,40.

Although persistent HR-HPV infection has been considered as the main cause of cervical progression 7-10,32, it is unclear whether the relationship between persistent same-type HR-HPV infection and cervical progression is different from that between persistent different-type HR-HPV infection and cervical progression. Our analysis results found that the increased risk for progression of normal cervix in women with persistent HR-HPV infection was mainly attributable to persistent same-type HR-HPV infection; persistent different-type HR-HPV infection, HR-HPV clearance, and HR-HPV acquisition were not significantly associated with the progression risk of normal cervix. Compared with the HR-HPV persistently negative group, the clearance group and the acquisition group, the risk for progression of normal cervix decreased gradually in the persistent HR-HPV infection group. The risk for progression of normal cervix in the persistent same-type HR-HPV infection group was 22.26, 8.21, 7.19, and 4.61 times greater than that of the HR-HPV persistently negative group, the clearance group, the acquisition group, and the persistent different-type HR-HPV infection group, respectively. Our findings indicated that persistent same-type HR-HPV infection was the leading cause of progression of normal cervix.

Several strengths need to be mentioned in this study. First, this is a prospective cohort that screened subjects from a population including 39,988 women. Second, the subjects were women with normal pathology screened from abnormal cervical cytology, and the effect of type-specific HR-HPV infection in their progression has been rarely studied. Last, we compared the cervical progression risk between the persistent same-type HR-HPV infection group and the persistent different-type HR-HPV infection group, which was rarely studied previously. There are also several limitations in our study. First, the follow-up period was 2 years, not long enough to observe the occurrence of cervical cancer. Second, we did not assess the association between persistent type-specific HR-HPV infection and the progression risk of normal cervix, as the number of women with persistent infection of a certain HR-HPV genotype was small. Third, it should be noted that some persistent HR-HPV infection might be the reinfection with these same genotypes, which would underestimated the relationship between HR-HPV persistence and cervical progression.

In conclusion, HPV16, 52, 58, 53 and 51 were the top five HR-HPV genotypes in this screening population; Women infected with HPV16, 31/33, 58, 51, and 53 were at high risk for progression of normal cervix; There were no significant difference between multiple HR-HPV infection and single HR-HPV infection in the progression of normal cervix; Same-type HR-HPV persistence was positively associated with the progression risk of normal cervix, but different-type HR-HPV persistence was not linked to cervical progression. The combination of genotype distribution and changes of HR-HPV infection and their potential role in the progression of women with normal cervical pathology and abnormal cytology could be of great significance to the implementation of more precise regional type-specific cervical lesion screening programs and the development of next generation of vaccines in the future. In the future, we plan to follow up this population at 5-year intervals to explore the long-term impact of type-specific HR-HPV infection on cervical progression in women with normal pathology and abnormal cytology.

## Figures and Tables

**Table 1:** Distribution of demographic characteristics between women enrolled into this study and women excluded at follow-up

Characteristics	Women enrolled in this study, n (%)	Women excluded at follow-up, n (%)	χ^2^	*P*
**Age (years)**			4.14	0.124
≤35	103 (8.4)	33 (12.2)		
36-50	550 (44.6)	112 (41.3)		
>50	579 (47.0)	126 (46.5)		
**Education level**			3.07	0.220
Primary school or less	207 (16.8)	53 (19.6)		
Middle-school	530 (43.0)	124 (45.8)		
High-school or more	495 (40.2)	94 (34.7)		
**Tobacco smoking**			0.46	0.605
Yes or ever	20 (1.6)	6 (2.2)		
Never	1212 (98.4)	265 (97.8)		
**Alcohol drinking**			0.47	0.512
Yes or ever	52 (4.2)	14 (5.2)		
Never	1180 (95.8)	257 (94.8)		
**Yearly income (¥RMB)**			0.20	0.900
≤10000	350 (28.4)	75 (27.7)		
(10000,30000)	646 (52.4)	141 (52.0)		
>30000	236 (19.2)	55 (20.3)		
**History of female sterilization**			1.98	0.173
Yes	409 (33.2)	78 (28.8)		
No	823 (66.8)	193 (71.2)		
**Family history of cancer**			0.26	0.641
Yes	112 (9.1)	22 (8.1)		
No	1120 (90.9)	249 (91.9)		

**Table 2:** Effect of baseline HR-HPV type-specific infection on cervical progression in women with normal histology but abnormal cervical cytology

Infection status of HR-HPV genotypes	n	Progression, n (%)	χ^2^	*P*	HR (95% CI)	aHR (95% CI)
HR-HPV negative	875	10 (1.1)			1	1
HR-HPV positive	357	22 (6.2)	25.25	<0.001	5.39 (2.55, 11.39)	5.31 (2.51, 11.21)
HPV16 positive	122	13 (10.7)	38.88	<0.001	9.32 (4.09, 21.26)	7.10 (3.05, 16.53)
HPV18 positive	18	1 (5.6)	2.82	0.202	4.86 (0.62, 37.97)	5.58 (0.69, 44.63)
HPV31/33 positive	30	3 (10.0)	16.07	0.008	8.75 (2.41, 31.79)	6.95 (2.01, 26.26)
HPV35 positive	14	0 (0.0)	0.16	1	NA	NA
HPV39 positive	27	0 (0.0)	0.31	1	NA	NA
HPV51 positive	33	2 (6.1)	5.89	0.067	5.30 (1.16, 24.20)	5.04 (1.06, 23.94)
HPV52 positive	72	3 (4.2)	4.49	0.069	3.65 (1.00, 13.25)	3.04 (0.83, 11.19)
HPV53 positive	37	2 (5.4)	4.97	0.082	4.73 (1.04, 21.59)	4.88 (1.06, 22.43)
HPV56 positive	22	0 (0.0)	0.25	1	NA	NA
HPV58 positive	65	5 (7.7)	16.53	0.002	6.73 (2.30, 19.69)	5.74 (1.94, 16.96)
HPV59 positive	11	0 (0.0)	0.13	1	NA	NA
HPV66 positive	19	0 (0.0)	0.22	1	NA	NA
HPV68 positive	17	0 (0.0)	0.20	1	NA	NA
Single HR-HPV infection	249	15 (6.0)	21.24	<0.001	5.27 (2.37, 11.73)	4.67 (2.07 10.55)
multiple HR-HPV infection	108	7 (6.5)	16.12	<0.001	5.67 (2.16, 14.90)	4.67 (1.74, 12.61)

HR: Hazard ratio; CI: confidence interval; aHR: ajusted oral contraceptive use and age.

**Table 3:** Comparison of effect of single and multiple HR-HPV infections on cervical progression in women with normal histology but abnormal cervical cytology

Genotypes of HR-HPV infection	n	Progression, n (%)	χ^2^	*P*	HR (95% CI)	aHR (95% CI)
Single HR-HPV	249	15 (6.0)			1	1
multiple HR-HPV	108	7 (6.5)	0.03	1	1.08 (0.44, 2.64)	1.21 (0.48, 3.04)
Single HPV16	77	7 (9.1)			1	1
Multiple HPV16	45	6 (13.3)	0.54	0.55	1.47 (0.49, 4.36)	1.37(0.46, 4.08)
Single HPV31/33	23	1 (4.3)			1	1
Multiple HPV31/33	7	2 (28.6)	3.5	0.13	6.57 (0.60, 72.47)	7.65 (0.63, 83.34)
Single HPV51	18	2 (11.1)			1	1
Multiple HPV51	15	0 (0.0)	1.77	0.49	NA	NA
Single HPV53	20	2 (10.0)			1	1
Multiple HPV53	17	0 (0.0)	1.8	0.49	NA	NA
Single HPV58	33	2 (6.1)			1	1
Multiple HPV58	32	3 (9.4)	0.25	0.67	1.55 (0.26, 9.26)	2.59 (0.33, 20.55)

HR: Hazard ratio; CI: confidence interval; aHR: adjusted oral contraceptive use and age.

**Table 4:** Association between changes of HR-HPV infection and cervical progression in women with normal histology but abnormal cervical cytology

Changes of HR-HPV infection	n	Progression, n (%)	χ^2^	*P*	OR (95% CI)	aOR (95% CI)
Persistently negative	762	7 (0.9)			1	1
Acquisition	113	3 (2.7)	2.63	0.128	2.94 (0.75, 11.54)	2.99 (0.74, 12.10)
Clearance	243	6 (2.5)	3.47	0.095	2.69 (0.90, 8.00)	2.12 (0.69, 6.51)
Persistently positive	114	16 (14.0)^abc^	66.73	<0.001	15.28 (6.29, 37.14)	13.96 (5.71, 34.10)
Persistent different-type infection	47	2 (4.3)	4.48	0.092	4.63 (0.96, 22.30)	3.92 (0.81, 18.99)
Persistent same-type infection	67	14 (20.9)^abcd^	99.54	<0.001	22.75 (9.18, 56.36)	22.26 (8.98, 55.17)

^a^There was a statistical difference in comparison to the group with HR-HPV persistently negative. ^b^There was a statistical difference in comparison to the group with HR-HPV newly acquired. ^c^There was a statistical difference in comparison to the group with HR-HPV clearance. ^d^There was a statistical difference in comparison to the group with persistent different-type HR-HPV infection. OR: odds ratio; CI: confidence interval; aOR: ajusted oral contraceptive use and age.

## Abbreviations

HPV: human papillomavirus
HR-HPV: high-risk HPV
LR-HPV: low-risk HPV
CIN: cervical intraepithelial neoplasia
CIN1: low-grade cervical intraepithelial neoplasia
CIN2/3: high-grade cervical intraepithelial neoplasia
CIN1+: CIN1 and above
CIN2+: CIN grade 2 and above
TCT: thinprep cytologic test
NILM: negative for intraepithelial lesion or malignancy
ASC-US+: atypical squamous cells of undetermined significance and above
HRs: Hazard ratios
aHR: adjusted HR
ORs: odds ratios
aOR: adjusted OR
CIs: confidence intervals
